# Air pollution and retinal vessel diameter and blood pressure in school-aged children in a region impacted by residential biomass burning

**DOI:** 10.1038/s41598-021-92269-x

**Published:** 2021-06-17

**Authors:** Jill Korsiak, Kay-Lynne Perepeluk, Nicholas G. Peterson, Ryan Kulka, Scott Weichenthal

**Affiliations:** 1 Department of Epidemiology, Biostatistics and Occupational Health, McGill University, 1100 Pine Avenue West, Montreal, QC H3A 1A3 Canada; 2 Air Health Science Division, Health Canada, 269 Laurier Ave West, Ottawa, ON K1A 0K9 Canada

**Keywords:** Epidemiology, Paediatric research, Risk factors

## Abstract

Little is known about the early-life cardiovascular health impacts of fine particulate air pollution (PM_2.5_) and oxidant gases. A repeated-measures panel study was used to evaluate associations between outdoor PM_2.5_ and the combined oxidant capacity of O_3_ and NO_2_ (using a redox-weighted average, O_x_) and retinal vessel diameter and blood pressure in children living in a region impacted by residential biomass burning. A median of 6 retinal vessel and blood pressure measurements were collected from 64 children (ages 4–12 years), for a total of 344 retinal measurements and 432 blood pressure measurements. Linear mixed-effect models were used to estimate associations between PM_2.5_ or O_x_ (same-day, 3-day, 7-day, and 21-day means) and retinal vessel diameter and blood pressure. Interactions between PM_2.5_ and O_x_ were also examined. O_x_ was inversely associated with retinal arteriolar diameter; the strongest association was observed for 7-day mean exposures, where each 10 ppb increase in O_x_ was associated with a 2.63 μm (95% CI − 4.63, − 0.63) decrease in arteriolar diameter. Moreover, O_x_ modified associations between PM_2.5_ and arteriolar diameter, with weak inverse associations observed between PM_2.5_ and arteriolar diameter only at higher concentrations of O_x_. Our results suggest that outdoor air pollution impacts the retinal microvasculature of children and interactions between PM_2.5_ and O_x_ may play an important role in determining the magnitude and direction of these associations.

## Introduction

Outdoor air pollution is associated with adverse cardiovascular outcomes^1,2^. Although cardiovascular disease (CVD) manifests in adulthood, preclinical changes that contribute to and accelerate the development of CVD begin in childhood^3^. Therefore, identifying early-life modifiable exposures that adversely affect cardiovascular health may provide important information to help prevent CVD in later life.

Most research on associations between ambient air pollution and cardiovascular outcomes has focused on particulate matter exposure and consistent evidence from epidemiological and animal studies support a causal relationship^1^. Oxidant gases, such as ozone (O_3_) and nitrogen dioxide (NO_2_), have also been associated with adverse cardiovascular outcomes, although results have been less consistent^4,5^. Individuals are exposed to both particulate matter and oxidant gases simultaneously, and some evidence suggests these pollutants interact to affect health outcomes. For example, stronger associations between long-term^6^ and short-term^7^ fine particulate matter air pollution (PM_2.5_) and mortality were found when the combined oxidant capacity of NO_2_ and O_3_ (using a redox-weighted average, O_x_) was higher, highlighting the importance of considering O_x_ when evaluating PM_2.5_ health effects.

The microcirculation represents a large component of the circulatory system and microvascular dysfunction is an important predictor of CVD events^8^. Measuring the structure of the retinal microvasculature through fundus photography can serve as a simple, non-invasive method to evaluate microvascular health^9^, as the retinal microcirculation is anatomically and physiologically similar to the cerebrovascular^10^ and coronary^11^ microcirculation. Of the various parameters that can be estimated with fundus photography, the most common and easily estimated parameters are the diameters of retinal blood vessels. The relationship between air pollution and retinal blood vessel diameter has been examined several times in adults in cross-sectional^12^ and repeated-measures studies^13–15^, and twice in children in repeated-measures studies^16,17^. In one study of school-aged children living in an urban centre in Belgium, short-term PM_2.5_ (measured on the same day as the retinal image and the day before) was associated with narrower retinal arteriolar diameter and wider venular diameter^16^. In another study of children ages 4–6 years (also living in Belgium), PM_2.5_ measured during the same day as the retinal image, the day before the retinal image, and the week before the retinal image was associated with both narrower and wider retinal arterial diameter, depending on the exposure lag, while NO_2_ was not associated with retinal vessel diameter^17^. Due to the limited number of studies that have explored these associations in children and inconsistent results, these relationships necessitate further exploration.

Another preclinical cardiovascular outcome that may be adversely affected by outdoor air pollution is blood pressure^4,5,18^, but associations between short-term air pollution and blood pressure have not been extensively studied in children. In a recent meta-analysis of four studies that looked at associations between short-term air pollution (defined as < 30 days) and blood pressure in children, each 10 μg/m^3^ increase in particulate matter < 10 μm (PM_10_) was associated with a very small (< 1 mm Hg) increase in systolic blood pressure, while no clear associations were observed between PM_10_ or PM_2.5_ and diastolic blood pressure^19^. An understanding of the relationship between air pollution and blood pressure in children is important because childhood blood pressure tracks into adulthood^20^ and elevated blood pressure is an important risk factor for the development of cardiovascular disease.

To our knowledge, no studies have explored how the combined oxidant capacity of NO_2_ and O_3_ (O_x_) affects retinal blood vessel diameter or blood pressure, or whether associations between PM_2.5_ and these health outcomes are modified by O_x_. In addition, no studies have focused specifically on the impact of residential biomass burning-related PM_2.5_ to changes in the retinal microvasculature or blood pressure. This is an important consideration because residential biomass burning is a major source of PM_2.5_ in rural Canada^21,22^ due to the prevalence of wood burning to heat homes, and biomass-burning sources of PM_2.5_ may be harmful to cardiovascular health^22^.

To address gaps in our current understanding of air pollution impacts on cardiovascular health of children, we conducted a panel study to examine associations between outdoor PM_2.5_ and O_x_ on changes to retinal vessel diameter and blood pressure in children living in a region of Canada known to be impacted by residential biomass burning. We also considered whether the impact of PM_2.5_ on retinal blood vessel diameter or blood pressure was modified by outdoor concentrations of O_x_.

## Materials and methods

### Study design and population

We conducted a repeated-measures panel study at two elementary schools in the neighbouring communities of Courtenay and Cumberland on the east coast of central Vancouver Island, in the province of British Columbia, Canada. The distance between the two schools is approximately 8 km. This is a rural area of Canada, with a population size of approximately 26,000 in Courtenay and 4,000 in Cumberland in 2016 (the most recent census year). The study took place from September 2018 to June 2019 in Courtenay, and from September 2019-March 2020 in Cumberland (the study was terminated three months early in Cumberland because of school closures due to the COVID-19 pandemic). The study took place over sequential school years (instead of at both schools in the same school year) because study equipment and research staff were limited. This area has elevated outdoor PM_2.5_ concentrations during the cold season (approximately November–April) because many households rely on wood burning as their primary heating source^22^. During the warmer season, outdoor PM_2.5_ concentrations are typically very low (i.e. < 5 μg/m^3^)^22^.

Children at each school were eligible to participate if they were 4–12 years of age at enrollment, lived in a non-smoking home, and resided in the community surrounding either school. Recruitment occurred during September of each school year, and health outcome measurements began in October. Exams were scheduled at intervals of approximately one month and were staggered throughout each month (as opposed to measuring everyone on the same day) in order to increase exposure variation and minimize the impact on regular school activities. Exams took place on Thursday and Friday mornings at the school site in Courtenay, and throughout the week in the morning and early afternoon in Cumberland. Oral assent was obtained from children and written informed consent was obtained from their parent/guardian. At baseline, parents/guardians of each participant completed a questionnaire to collect basic sociodemographic and household information. The study was approved by McGill University Research Ethics Board and the Health Canada Research Ethics Board and all methods were performed in accordance with the relevant guidelines and regulations.

### Air pollutants and meteorological data

In the first year of the study, daily mean outdoor PM_2.5_ concentrations in Courtenay were measured using a BAM (Beta-Attenuation Monitor) 1020 instrument located at the provincial air monitoring station situated on the playground of the school. In case there were any problems or gaps in data collection with the government-run monitor, we also set up a Partisol 2025i sequential air sampler at the same location, which collected daily integrated PM_2.5_ samples that were subsequently sent for gravimetric analysis. However, for this year of the study, we ended up only using PM_2.5_ measurements from the BAM instrument in our analyses because there were fewer missing data. In the second year of the study in Cumberland, the school was not located at a provincial monitoring station so PM_2.5_ was only measured using a Partisol 2025i sequential air sampler that we set up on the roof of the school. Although the PM_2.5_ values used in analysis were from different instruments each year of the study, we observed a strong correlation in duplicate measurements in Courtenay (r^2^ = 0.94) and both instruments are considered acceptable methods to monitor PM_2.5_ by the United States Environmental Protection Agency^23^.

For both years of the study, ozone and nitrogen dioxide were measured at the provincial air monitoring site in Courtenay with an API T400 UV Absorption O_3_ analyzer and an API T200 chemiluminescence NO/NO_2_/NO_x_ analyzer, respectively; due to equipment limitations, we were unable to set up our own monitors for O_3_ and NO_2_ in Cumberland so relied on measurements from Courtenay as approximations. The combined weighted oxidant capacity (O_x_) of NO_2_ and O_3_ was calculated as a weighted average of NO_2_ and O_3_, with weights equivalent to the respective redox potentials using the formula O_x_ = [(1.07 × NO_2_) + (2.075 × O_3_)]/3.145), as previously described^24,25^. Indoor air pollution was not measured in this study. Meteorological data, including mean daily temperature, wind speed, precipitation, and humidity were available from a provincial monitoring station located approximately 8 km from the school in Courtenay and 15 km from the school in Cumberland.

In the second year of the study (in Cumberland), there were some days with missing PM_2.5_ data due to a delay in setting up the PM_2.5_ monitor at the start of the study and occasional technical issues throughout the study. A model to predict missing PM_2.5_ was developed, and predicted values were used to impute missing PM_2.5_. The prediction model regressed log-transformed PM_2.5_ on several predictors including same-day PM_2.5_, NO_2_, temperature, wind speed, and precipitation measured at a nearby provincial monitoring station. Global search regression using the *gsreg* command in Stata was used to select the final prediction model, considering all possible combinations of interactions and square terms of predictor variables. The best fitting model had a R^2^ of 0.72. There was a total of 58 days in which PM_2.5_ was imputed (approximately 12% of PM_2.5_ values in the time series).

### Clinical exams

Clinical exams were conducted by two trained research assistants (one research assistant at each site) and involved imaging the retinal microvasculature and measuring blood pressure, height, and weight. All exams took place in a designated, quiet room in each school.

The fundus of the left and right eye of participants was photographed with a Canon CR2-AF 45° 20.2-megapixel digital nonmydriatic retinal camera in a darkened room. Images were analyzed by one grader (J.K.) using the semi-automatic MONA-REVA software (version 3.0.0, VITO Health, Mol, Belgium). For each participant, images from either the left or right eye were analyzed; the choice of whether to analyze the left or right eye of each participant depended on which eye had the most high quality images (where image quality was judged by how sharp the image was, whether the optic disc was centered, and whether the arterioles and venules were distinguishable from one another). Epidemiological studies have demonstrated a high correlation in retinal vessel diameters between the left and right eye^26,27^. When analyzing the images, the diameter of the optic disc was first determined, then the width of the retinal arterioles and venules were measured within an area equal to 0.5–1 times the disc diameter from the optic disc margin (Figure S1 in the Supplemental Material). Diameters of the 6 largest arterioles and venules were used in the revised Parr Hubbard formula^28^ to estimate Central Retinal Arteriolar Equivalent (CRAE) and Central Retinal Venular Equivalent (CRVE), summary measures reflecting average arteriolar and venular diameter. For each participant, the same 6 arterioles and venules were used to calculate CRAE and CRVE in repeated measurements.

Following fundus photography, blood pressure was measured with the SunTech CT40 vital signs device. While sitting upright in a chair with their non-dominant arm resting on a table, an appropriately sized arm cuff was selected based on the circumference of the child’s upper arm, and blood pressure was measured twice with one minute between each reading. If systolic or diastolic blood pressure from the two successive readings were > 10 mm Hg apart, a third reading was done. The average of the two closest readings was calculated and used for analysis.

With shoes and bulky clothing removed, height was measured to the nearest 0.1 cm with the Seca 213 Stadiometer, and weight was measured to the nearest 0.1 kg using the Seca 874 Digital Scale. Measurements were taken in duplicate, and an average was calculated. Body mass index-for-age z-scores were then calculated based on the World Health Organization child growth standards^29^.

### Statistical analyses

#### Associations between outdoor air pollution and retinal blood vessel diameter

Linear mixed-effect models with a random subject intercept (with a first order autoregressive correlation structure) were used to evaluate associations between PM_2.5_ (as a continuous variable, in units of μg/m^3^) or O_x_ (a continuous variable, in units of ppb) and within-person changes in CRAE or CRVE (continuous variables, in units of μm). We assessed associations between CRAE or CRVE with four different exposure lags: PM_2.5_ or O_x_ on the day of the retinal image, 3-day mean (mean of PM_2.5_ or O_x_ on the day of the retinal image and two preceding days), 7-day mean, and 21-day mean. These time periods were selected to examine both acute and sub-chronic exposures. For each exposure-outcome relationship, we ran crude models, and models adjusted for an a priori list of potential confounders or predictors of retinal blood vessel diameter, including 7-day mean temperature (degrees Celsius) and humidity (%) (which may be correlated with seasonal differences in air pollution concentrations), body mass index-for-age z-score at the time of the retinal image, sex, age (years), highest level of maternal education (high school or less/ community or technical college/ university), and time of day of outcome assessment (≤ 11:00 AM or > 11:00 AM). We also explored whether associations between PM_2.5_ and retinal vessel diameter were modified by concentrations of O_x_ by running models with an interaction term between PM_2.5_ and O_x_ (as continuous variables using the same exposure lag for both air pollutants)_,_ while adjusting for the same set of covariates identified above. A p-value less than 0.05 for the interaction term was interpreted as evidence of effect modification (on the additive scale). We explored whether including a fixed effect for school was necessary to account for potential clustering within schools, but it did not improve model fit based on the minimum Akaike Information Criterion (AIC) so was not included in the final models. We also explored potential non-linear relationships between continuous covariates and CRAE or CRVE using spline terms, but as splines did not improve model fit (based on the minimum AIC), final models included linear terms for all continuous covariates. Residual plots were generated to verify model assumptions. All estimates are expressed as a change in retinal arteriolar or venular diameter per 5 μg/m^3^ increase PM_2.5_ or 10 ppb increase in O_x_, which reflect the approximate interquartile ranges of PM_2.5_ and O_x_.

#### Associations between outdoor air pollution and blood pressure

Linear mixed-effect models with a random subject intercept (and a first order autoregressive correlation structure) were used to evaluate associations between short-term and sub-chronic PM_2.5_ or O_x_ (the same exposure lags described above) and systolic and diastolic blood pressure. Similar to analyses for retinal vessel diameter, crude models, adjusted models (including the same set of covariates identified above), and models with an interaction term between PM_2.5_ and O_x_ were examined.

#### Sensitivity analyses

Several sensitivity analyses were conducted. First, analyses were repeated excluding retinal images or blood pressure measurements in which the relevant PM_2.5_ exposure lags included imputed PM_2.5_ values. Second, instead of evaluating associations between O_x_ and retinal blood vessel diameter and blood pressure, we looked at associations with each gas (O_3_ or NO_2_) individually. Third, we additionally adjusted our models for season (fall/winter/spring/summer).

All data cleaning and manipulation were conducted using Stata v.15 (StataCorp, College Station, TX), and all modelling was conducted using R (R-project.org).

## Results

### Study population

A description of the study population is presented in Table 1. A total of 71 children (median age of 8 years) enrolled in the study and high-quality retinal images were available for 64 of these children. Most participants (N = 54, 76%) enrolled during the second year (2019–2020) of the study. The sample was predominantly Caucasian (N = 64, 90%), there were a similar number of boys and girls, and most mothers of participants had some post-secondary education. The majority of participants lived in households that used electricity (N = 46, 65%) or natural gas (N = 21, 30%) as their primary heating source, while few households used wood burning as their primary heating source (N = 3, 4%). The use of woodstoves or wood fireplaces as a secondary source of heating was uncommon in this sample (N = 6, 8%), and 17 participants (24%) lived in households that used an air filter. The average (± standard deviation) body mass index-for age z-score was 0.7 ± 1.3, indicating body mass index of children was slightly higher than the age and sex-specific reference population. Mean (± standard deviation) systolic and diastolic blood pressure at baseline were 106 ± 7 and 63 ± 5 mm Hg, respectively, while mean (± standard deviation) CRAE and CRVE at baseline were 181.51 ± 11.88 and 260.34 ± 15.70 μm.

**Table 1:** Description of the study population.

**Socio-demographic characteristics**	
Total enrolled participants, N	71
Participants with retinal images available^a^, N	64
**Date on study, n (%)**	
September 2018–June 2019	17 (24)
September 2019–March 2020	54 (76)
Age (years) at baseline, median (range)	8 (4–12)
Girls, n (%)	33 (46)
Caucasian, n (%)	64 (90)
**Highest level of maternal education complete, n (%)**	
Graduated high school or less	11 (15)
Some or graduated community/technical college	15 (21)
Some or graduated university	45 (63)
**Household characteristics**	
**Main heating source in home, n (%)**	
Wood	3 (4)
Natural gas	21 (30)
Electricity	46 (65)
Oil	1 (1)
Use of a woodstove or wood fireplace in home as a secondary heating source^b^, n (%)	6 (8)
Use of air filter in home, n (%)	17 (24)
**Cardiovascular measures**	
Central retinal arteriolar equivalent (μm), mean ± SD	181.51 ± 11.88
Central retinal venular equivalent (μm), mean ± SD	260.34 ± 15.70
Systolic blood pressure (mm Hg), mean ± SD	106 ± 7
Diastolic blood pressure (mm Hg), mean ± SD	63 ± 5
Body mass index-for-age z-score^c^, mean ± SD	0.7 ± 1.3

^a^High-quality images were unavailable for some participants due to blinking, inability to sit still, and general discomfort with getting their eyes photographed.

^b^Excludes participants in which a woodstove/wood fireplace is the main source of heating.

^c^Body mass index-for-age-and-sex z-score calculated based on World Health Organization growth charts.

There was a total of 344 high quality retinal images and 432 blood pressure measurements. The median number of retinal images and blood pressure measurements per child was 6 but some children had as few as three measurements. The maximum number of retinal images was 6 per child, and for blood pressure the maximum number of measurements was 10 per child. Median time between retinal images and blood pressure measurements was 28 days (range 20–63 days).

### Exposure characteristics

**Figure 1:**
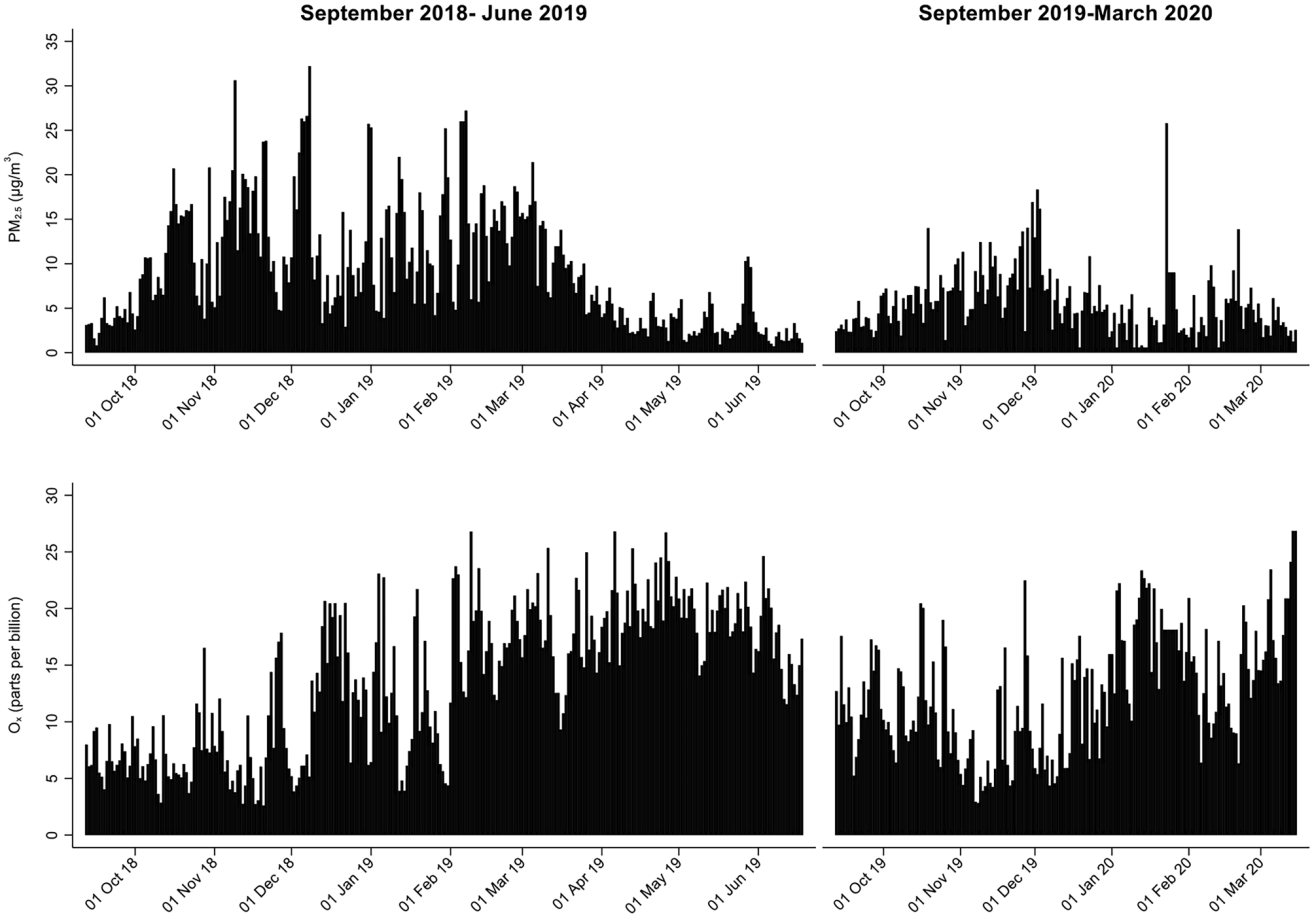
Distribution of daily mean ambient PM_2.5_ (μg/m^3^) and O_x_ (parts per billion) over the study duration.

Distributions of daily mean outdoor PM_2.5_ and O_x_ concentrations throughout the study are shown in Fig. 1 and additional exposure characteristics are provided in Table S1 of the Supplemental Material. Overall, mean daily PM_2.5_ ranged from < 1 μg/m^3^ to 32 μg/m^3^ over the entire study period, and was slightly higher and more variable in the first year of the study (mean ± standard deviation: 9 ± 7 μg/m^3^) than in the second year of the study (mean ± standard deviation: 6 ± 4 μg/m^3^). Average PM_2.5_ on the day of the retinal image was the same as the 3-day mean, 7-day mean, and 21-day mean concentrations (7 μg/m^3^), although the standard deviation was slightly larger on the day of the retinal image (standard deviation: 6 μg/m^3^) compared to the 3-day and 7-day means (standard deviation of 4 μg/m^3^ for both lags), and the 21-day mean (standard deviation: 3 μg/m^3^). O_x_ ranged from 3 to 27 ppb over the entire study period, and was slightly higher and more variable during the first year of the study (mean ± standard deviation: 14 ± 6 ppb) compared to the second year of the study (mean ± standard deviation: 13 ± 5 ppb). Mean O_x_ for all exposure lags was 13 ppb, and the standard deviation was slightly larger on the day of the retinal image (6 ppb) compared to the 3-day, 7-day, and 21-day means (5 ppb). There was a moderate inverse correlation between PM_2.5_ and O_x_ based on Pearson's correlation coefficient (r^2^ = − 0.43).

#### Associations between outdoor PM_2.5_ or O_x_ and retinal blood vessel diameter

**Figure 2:**
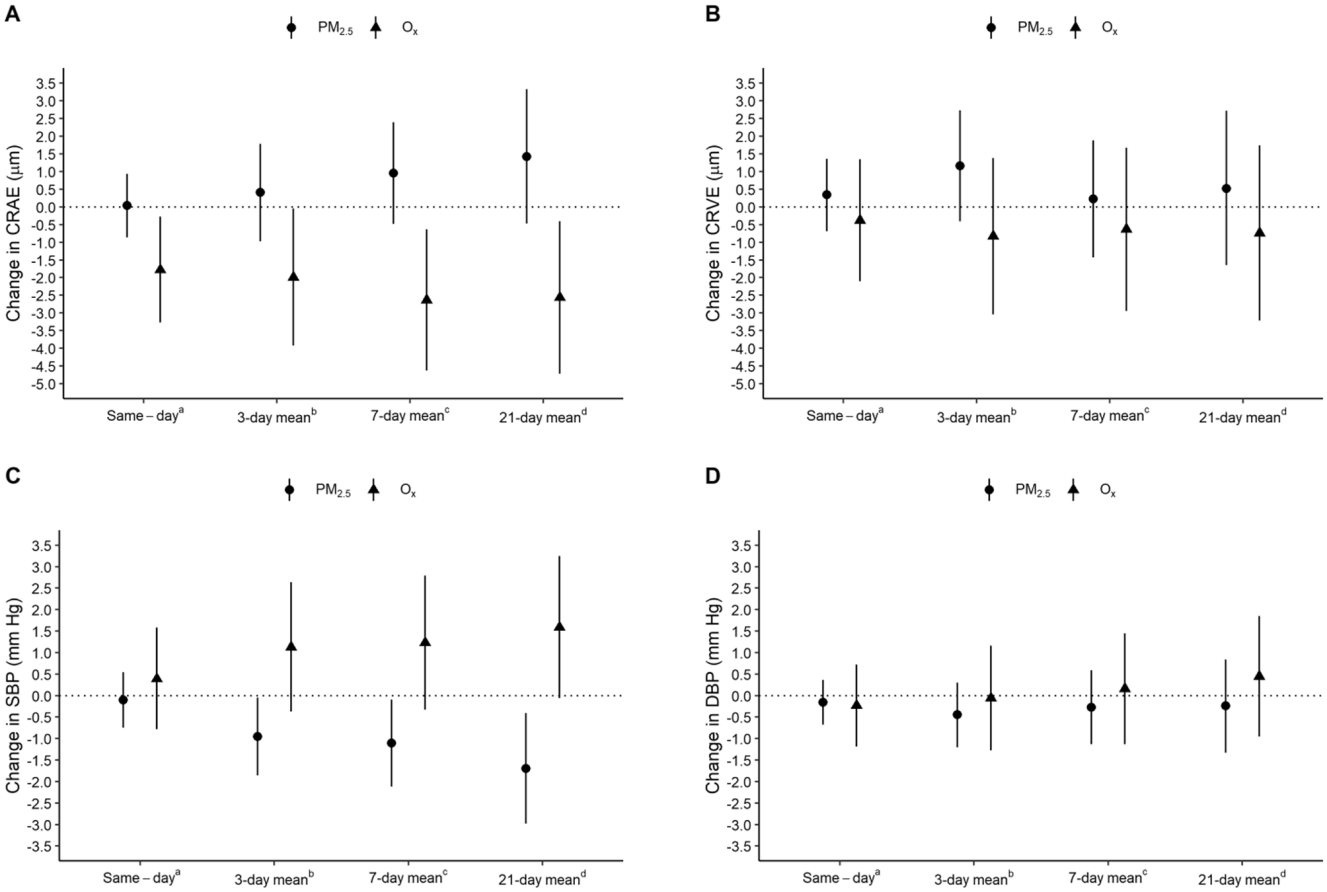
Estimated change (95% confidence interval) in (**A**) central retinal arteriolar diameter (CRAE, μm); (**B**) central retinal venular diameter (CRVE, μm); (**C**) systolic blood pressure (SBP, mm Hg) and; (**D**) diastolic blood pressure (DBP, mm Hg) per 5 μg/m^3^ increase in PM_2.5_ or 10 ppb increase in O_x_. Models adjusted for 7-day mean temperature and humidity, body mass index-for-age z-score on the day of the retinal image, sex, age (years), maternal education (high school or less vs. community/technical college vs. university), and time of day of outcome assessment (≤ 11:00 AM vs. > 11:00 AM). ^a^PM_2.5_ or O_x_ on the same day as the outcome assessment. ^b^Mean PM_2.5_ or O_x_ on the day of the outcome assessment and two preceding days. ^c^Mean PM_2.5_ or O_x_ on the day of the outcome assessment and 6 preceding days. ^d^Mean PM_2.5_ or O_x_ on the day of the outcome assessment and 20 preceding days.

Associations between PM_2.5_ or O_x_ from single-pollutant models and retinal arteriolar and venular diameter are presented in Fig. 2 and Tables S2 and S3 of the Supplemental Material. In adjusted models, PM_2.5_ was associated with a small increase in CRAE but 95% confidence intervals included the null. The strength of this association was largest for the 21-day exposure lag: a 5 μg/m^3^ increase in 21-day mean PM_2.5_ was associated with a 1.42 μm increase in CRAE (95% CI − 0.47, 3.32). On the other hand, O_x_ was consistently associated with a reduction in CRAE and the strongest association was for the 7-day exposure lag: a 10 ppb increase in O_x_ was associated with a 2.63 μm decrease in CRAE (95% CI − 4.63, − 0.63).

In general, positive association were observed between PM_2.5_ and venular diameter and inverse associations were observed between O_x_ and venular diameter but the strength of these associations was small and 95% confidence intervals included the null in all adjusted models. There were no notable differences in associations between PM_2.5_ and CRAE or CRVE when analyses excluded retinal images with imputed PM_2.5_ (Table S4 of the Supplemental Material). In sensitivity analyses, estimated associations between O_3_ and retinal blood vessel diameter were similar to that of O_x_ (Table S5 of the Supplemental Material), while NO_2_ was positively associated with retinal arteriolar and venular diameter, but estimates were imprecise and all confidence intervals included the null (Table S6 of the Supplemental Material). When models were additionally adjusted for season, conclusions remain the same (Table S7 and S8 of the Supplemental Material).

**Figure 3:**
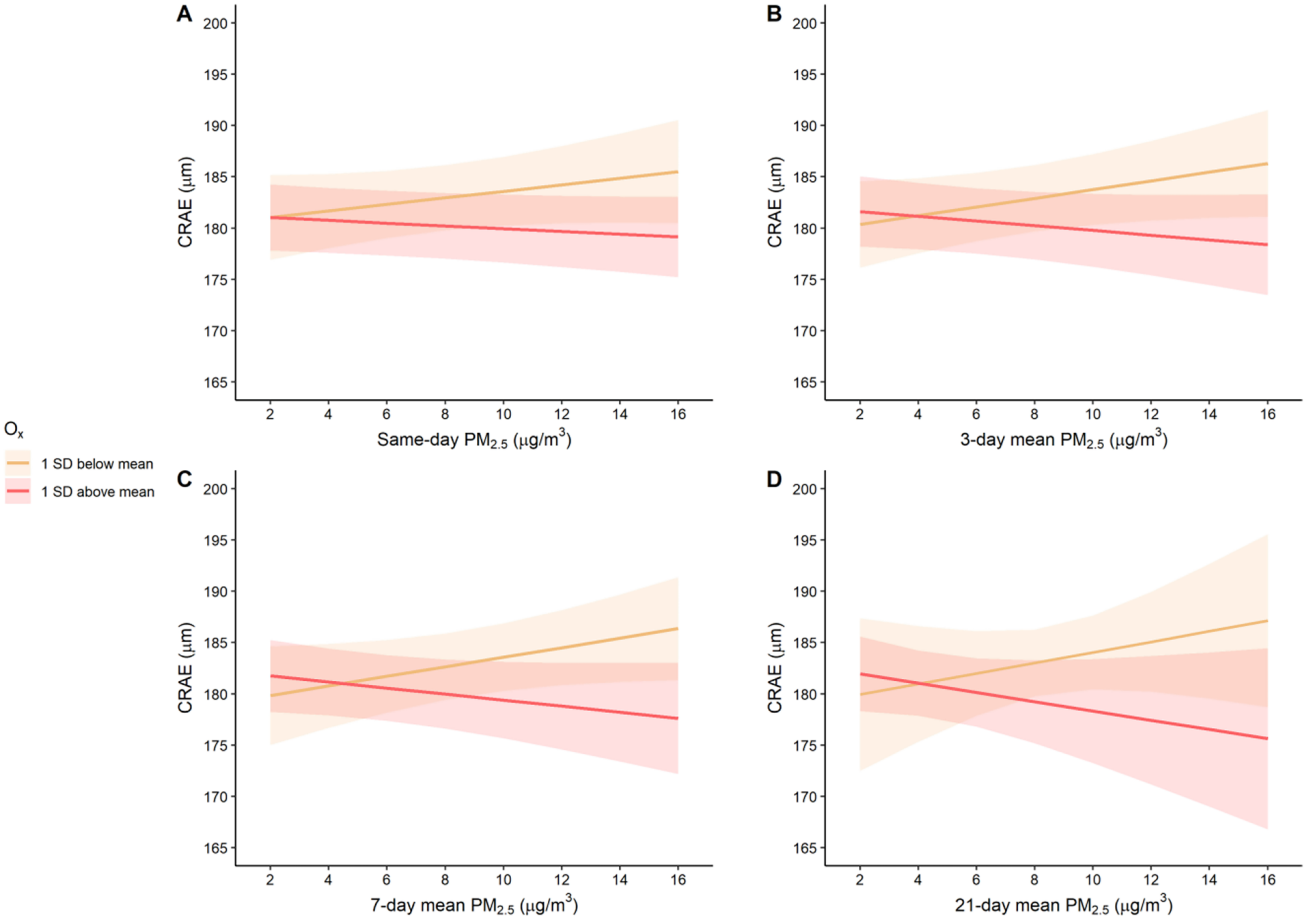
Predicted values and 95% confidence intervals for central retinal arteriolar equivalent (CRAE) at different concentrations of PM_2.5_, stratified by O_x_ (1 standard deviation below and above mean O_x_). Plots correspond to (**A**): Same-day exposure lag; (**B**): 3-day mean exposure lag; (**C**): 7-day mean exposure lag; (**D**): 21-day mean exposure lag.

Models including an interaction term between PM_2.5_ and O_x_ suggested that O_x_ modified associations between outdoor PM_2.5_ and retinal arteriolar diameter (p-values from interaction terms for same-day, 3-day mean, 7-day mean and 21-day mean exposures: 0.10, 0.04, 0.02, and 0.03, respectively). To visualize the associations between PM_2.5_ and CRAE modified by O_x_, we plotted predicted values of CRAE across a range of PM_2.5_ concentrations (2–16 μg/m^3^) stratified by O_x_ concentrations 1 standard deviation above or below the mean (Fig. 3). This figure suggests that when O_x_ is low there is a weak positive association between PM_2.5_ and CRAE, while when O_x_ concentrations are higher there is a weak inverse association between PM_2.5_ and CRAE. These trends were more pronounced in the 3-day, 7-day, and 21-day lags compared to same-day exposure. Similar figures were generated to visualize how concentrations of PM_2.5_ modified the associations between O_x_ and CRAE and suggest that a negative association between O_x_ and CRAE is only present when concentrations of PM_2.5_ were high (i.e., 1 standard deviation above the mean) (Figure S2 of the Supplemental Material). There was no evidence of interaction between PM_2.5_ and O_x_ for CRVE (p-values from interaction terms for same-day, 3-day mean, 7-day mean, and 21-day mean exposures: 0.52, 0.63, 0.14, and 0.83, respectively).

### Associations between outdoor PM_2.5_ or O_x_ and blood pressure

Associations between outdoor PM_2.5_ or O_x_ concentrations and blood pressure are presented in Fig. 2 and Tables S2 and S3 of the Supplemental Material. In adjusted models, each 5 μg/m^3^ increase in 3-day mean PM_2.5_ was associated with a 0.95 mm Hg reduction in systolic blood pressure (95% CI − 1.86, − 0.05), 7-day mean PM_2.5_ was associated with a 1.11 mm Hg reduction in systolic blood pressure (95% CI − 2.12, − 0.09), and 21-day mean PM_2.5_ was associated with a 1.70 mm Hg reduction in systolic blood pressure (95% CI − 2.98, − 0.41), but these associations were slightly attenuated and 95% confidence intervals included the null in sensitivity analyses excluding exams where PM_2.5_ was imputed (Table S4 of the Supplemental Material). Conversely, positive associations were observed between O_x_ and systolic blood pressure, with the largest association detected for the 21-day exposure lag (estimated change per 10 ppb increase in 21-day mean O_x_ from an adjusted model: 1.59 (95% CI − 0.06, 3.25)), but confidence intervals included the null for all exposure lags. There were no clear associations between PM_2.5_ or O_x_ and diastolic blood pressure. In sensitivity analyses, associations between O_3_ and blood pressure were similar to those found for O_x_ and no clear relationship was observed between NO_2_ and blood pressure (Tables S5 and S6 of the Supplemental Material). When models were additionally adjusted for season, conclusions are similar except the confidence intervals for associations between 3-day and 7-day mean PM_2.5_ now include the null (Table S7 and S8 of the Supplemental Material).

There was evidence that 7-day mean O_x_ modified the associations between 7-day mean PM_2.5_ and systolic blood pressure (p-value from interaction term: 0.04), but there was no evidence of a significant interaction for the same-day, 3-day mean, or 21-day mean exposures (p-values from interaction terms for same-day, 3-day mean, and 21-day mean exposures: 0.63, 0.26, 0.55). Figure S3 in the Supplemental Material suggests that an inverse relationship between 7-day mean PM_2.5_ and systolic blood pressure is present when 7-day mean O_x_ concentrations are above average (i.e., 1 standard deviation above the mean), while there is no association when O_x_ concentrations are lower (i.e., 1 standard deviation below mean). O_x_ did not modify associations between PM_2.5_ or diastolic blood pressure for any exposure lags (p-value for interaction term for same-day, 3-day mean, 7-day mean, and 21-day mean exposures: 0.57, 0.46, 0.51 and 0.61).

## Discussion

Our findings suggest that outdoor air pollution in a region impacted by residential biomass burning has a measurable impact of the microvasculature of school-age children. Specifically, O_x_ was consistently associated with retinal arteriolar narrowing in single-pollutant models. Our findings also suggest that an important interaction may exist between outdoor concentrations of oxidant gases and PM_2.5_, as PM_2.5_ was only associated with arteriolar narrowing when O_x_ concentrations were elevated. We also found inverse associations between PM_2.5_ and systolic blood pressure and evidence of effect modification by O_x_ for the 7-day exposure lag, while in single-pollutant models there were trends towards positive associations between O_x_ and systolic blood pressure. No clear associations between PM_2.5_ or O_x_ and retinal venular diameter or diastolic blood pressure were observed.

Although this study did not conduct any source apportionment of PM_2.5_, it is known that residential biomass burning affects air quality in this region of Canada. For example, Hong et al.^30^ developed an algorithm that was applied to 23 communities in British Columbia, Canada, to identify smoky vs. non-smoky days, and classified 30% of days in Courtenay between 2014–2016 as smoky, making it the second smokiest community of the 23 studied. Moreover, Weichenthal et al.^22^ identified biomass burning as a major contributor to ambient PM_2.5_ in Courtenay by measuring daily levoglucosan (a tracer of biomass burning) levels from January 2014–March 2015. Furthermore, traffic-related air pollution is very minimal in this region because it is a rural location on an island with a small population size, and there are no major industries in the area that would affect air quality.

The biological mechanisms underlying air pollution impacts on the microcirculation and blood pressure are thought to be related to oxidative stress, inflammation, and disturbances to the autonomic nervous system^1,31^. Inhaled particles can stimulate the generation of reactive oxygen species causing both pulmonary and systemic oxidative stress and inflammation which contributes to endothelial dysfunction and vasoconstiction^1^. Arteriolar narrowing may contribute to elevated blood pressure because arterioles are the main regulators of peripheral blood flow and are essential in the maintenance of blood pressure^32^. In addition, air pollution exposure may lead to an imbalance of the autonomic nervous system which favours sympathetic pathways, and can contribute to endothelial dysfunction, vasoconstriction, and elevated blood pressure^1^.

In general, existing evidence from observational studies related to the associations between outdoor air pollution and blood pressure in children is inconsistent. For example, Yang et al.^33^ found that short-term exposure to PM_2.5_ was associated with very small increases in both systolic and diastolic blood pressure (< 1 mm Hg increase in systolic and diastolic blood pressure per 10 μg/m^3^ PM_2.5_) in a large study of approximately 190,000 children in China, but a smaller study in the Netherlands found no clear associations between short-term PM_10_, NO_2_ or O_3_ and systolic or diastolic blood pressure^34^. In another study in Belgium, consistent positive associations were detected between ultrafine particles and systolic blood pressure in children, but trends of an inverse association was observed for PM_2.5_^35^_._ Inverse associations between systolic blood pressure and short-term particulate matter^36,37^ and ozone^38^ have also been observed in adult populations. We are not sure why we observed inverse associations between air pollution and systolic blood pressure because our existing knowledge of physiological responses to air pollution generally would support positive associations^1^; however, these inconsistent findings highlight uncertainty in our current understanding of air pollution impacts on cardiovascular health. In this study, although we found limited evidence of effect modification by O_x_ for the associations between PM_2.5_ and blood pressure, it still is possible that complex interactions between air pollutants exist and contribute to the heterogeneity of results observed between studies.

Regarding the retinal microvasculature, previous evidence in adults^12,13^ and children^16^ have observed arteriolar narrowing in response to PM_2.5_ exposure. For example, Provost et al. found that same-day residential outdoor PM_2.5_ was associated with a 0.62 μm decrease in retinal arteriolar diameter (95% CI − 1.12, − 0.12) per 10 μg/m^3^ increase in PM_2.5_ in school-aged children in Belgium^16^. However, a second study by Luyten et al. found that the direction of associations between PM_2.5_ and retinal arteriolar diameter in children was sensitive to the exposure lag that was selected^17^. Results for retinal venular diameter have been less conclusive but tend to suggest positive associations with air pollution^16,17^. To our knowledge, no studies to date have examined associations between O_x_ or O_3_ and retinal blood vessel diameter but Luyten et al.^17^ investigated the impact of NO_2_ and did not find any clear associations.

The most interesting finding in our study is the interaction observed between PM_2.5_ and O_x_ in models for retinal arteriolar diameter. Specifically, the direction of the association between PM_2.5_ and arteriolar diameter was modified by concentrations of O_x_, with weak positive associations observed at lower concentrations of O_x_ and inverse associations observed at higher concentrations of O_x_. Similarly, the inverse association between O_x_ and retinal arteriolar diameter was only observed when concentrations of PM_2.5_ were high. This modifying role of O_x_ in PM_2.5_ health effects has been observed previously for other outcomes. For example, Weichenthal et al.^6^ found stronger associations between PM_2.5_ and all-cause, cardiovascular, and respiratory mortality when concentrations of O_x_ were higher, while Lavigne et al.^7^ observed similar results with short-term PM_2.5_ and all-cause and cardiovascular mortality. Together, this evidence highlights the importance of considering O_x_ when evaluating the health impacts of PM_2.5_ and also suggests possible co-benefits of regulatory interventions aimed at reducing outdoor air pollution (i.e. reducing O_x_ may also reduce the health impacts of PM_2.5_ even if PM_2.5_ mass concentrations remain unchanged).

Existing evidence suggests several possible mechanisms underlying the observed interaction between PM_2.5_ and O_x_. First, elevated ozone depletes antioxidants in the epithelial lining fluid of the respiratory tract^39^, and this may lower our defenses against reactive oxygen species produced in response to PM_2.5_ exposure, contributing to greater oxidative stress. In addition, ozone has been shown to increase the permeability of the lung epithelial barrier^40–42^, which may contribute to greater absorption of particles into the systemic circulation and greater health impacts of PM_2.5_. Lastly, oxidant gases can increase the toxicity of PM_2.5_ through photochemical aging processes; for example, exposure to ozone has been shown to increase the oxidative potential of particles from both engine exhaust^43,44^ and biomass burning^45^.

There are several strengths of this study, including the repeated measures design that eliminates potential confounding by variables that do not change within individuals over a short time period, exposure information for multiple air pollutants, and the study setting that allowed us to evaluate air pollution primarily from residential biomass burning. However, this study also had limitations. Foremost, this study is subject to non-differential, Berkson-type exposure measurement error because true personal PM_2.5_ or O_x_ exposures may differ from outdoor concentrations. The result of Berkson measurement error is a reduction in precision without any systematic bias^46^. Another limitation is we are evaluating short-term changes in retinal blood vessel diameter but how this may impact future health is not clear. We (and others^47^) hypothesize that repeated short-term damage to microvascular structure can lead to chronic microvascular changes in later life, but there are no longitudinal studies demonstrating this. In addition, there is likely some classical measurement error in estimating arteriolar and venular diameter, but this is almost certainly non-differential with respect to outdoor air pollution concentrations.

## Conclusion

In summary, these results suggest that short-term and sub-chronic exposures to air pollution impact the retinal microvasculature and blood pressure of children, and highlight the importance of considering potential interactions between air pollutants when evaluating cardiovascular health impacts. Given the small number of studies that have investigated the impact of outdoor air pollution on the retinal microvasculature or blood pressure in children, additional work is needed to confirm these findings.

## Supplementary Information

Supplementary Information.

## Abbreviations

AIC: Akaike information criterion
CI: Confidence interval
CRAE: Central retinal arteriolar equivalent
CRVE: Central retinal venular equivalent
CVD: Cardiovascular disease
DBP: Diastolic blood pressure
NO_2_: Nitrogen dioxide
O_3_: Ozone
O_x_: The combined redox-weighted oxidant capacity of NO_2_ and O_3_
PM_2.5_: Fine particulate matter air pollution
PM_10_: Particulate matter < 10 μm
SD: Standard deviation
SBP: Systolic blood pressure
