# Supplementary material for: Air pollution and retinal vessel diameter and blood pressure in school-aged children in a region impacted by residential biomass burning
Source: Sci Rep. 2021 Jun 17;11:12790. doi: 10.1038/s41598-021-92269-x (PMC8211781; doi:10.1038/s41598-021-92269-x)
Supplement: Supplementary file 1 — Supplementary Information. [file 41598_2021_92269_MOESM1_ESM.docx]

**SUPPLEMENTAL MATERIAL**

**Air Pollution and Retinal Vessel Diameter and Blood Pressure in School-Aged Children in a Region Impacted by Residential Biomass Burning**

Jill Korsiak, Kay-Lynne Perepeluk, Nicholas G. Peterson, Ryan Kulka, Scott Weichenthal

**Table S1:** PM_2.5_ and O_x_ exposure characteristics

|  | **Mean ± standard deviation** | **Median (range)** |
| --- | --- | --- |
| **PM_2.5_ (μg/m^3^)** |  |  |
| Over the entire study duration (September 2018-March 2020) | 8 ± 6 | 6 (<1- 32) |
| Year 1: September 2018-June 2019 | 9 ± 7 | 8 (<1- 32) |
| Year 2: September 2019-March 2020 | 6 ± 4 | 5 (<1- 26) |
| Same-day^a^ | 7 ± 6 | 6 (<1- 31) |
| 3-day mean^b^ | 7 ± 4 | 6 (1- 26) |
| 7-day mean^c^ | 7 ± 4 | 6 (2- 21) |
| 21-day mean^d^ | 7 ± 3 | 5 (3- 17) |
| **O_x_ (parts per billion)** |  |  |
| Over the entire study duration (September 2018-March 2020) | 13 ± 6 | 13 (3- 27) |
| Year 1: September 2018-June 2019 | 14 ± 6 | 15 (3- 27) |
| Year 2: September 2019-March 2020 | 13 ± 5 | 12 (3- 27) |
| Same-day^a^ | 13 ± 6 | 13 (3- 27) |
| 3-day mean^b^ | 13 ± 5 | 14 (4- 23) |
| 7-day mean^c^ | 13 ± 5 | 13 (4- 22) |
| 21-day mean^d^ | 13 ± 5 | 13 (6- 21) |

^a^ PM_2.5_ or O_x_ on the day of the outcome assessment

^b^ Mean PM_2.5_ or O_x_ on the day of the outcome assessment and two preceding days

^b^ Mean PM_2.5_ or O_x_ on the day of the outcome assessment and 6 preceding days

^d^ Mean PM_2.5_ or O_x_ on the day of the outcome assessment and 20 preceding days

**Table S2:** Estimated change and 95% confidence interval in retinal blood vessel diameter (μm) and blood pressure (mm Hg) associated with 5 μg/m^3^ increase in PM_2.5_

|  | **Crude** | **Adjusted^a^** |
| --- | --- | --- |
| **Retinal Blood Vessel Diameter** | | |
| ***Central retinal arteriolar diameter*** | |  |
| Same-day PM_2.5_^b^ | -0.09 (-0.94, 0.76) | 0.04 (-0.86, 0.93) |
| 3-day mean PM_2.5_^c^ | 0.20 (-1.03, 1.42) | 0.41 (-0.97, 1.78) |
| 7-day mean PM_2.5_^d^ | 0.64 (-0.64, 1.93) | 0.95 (-0.48, 2.39) |
| 21-day mean PM_2.5_^e^ | 0.94 (-0.63, 2.52) | 1.42 (-0.47, 3.32) |
| ***Central retinal venular diameter*** | |  |
| Same-day PM_2.5_^b^ | 0.49 (-0.49, 1.48) | 0.34 (-0.69, 1.36) |
| 3-day mean PM_2.5_^c^ | 0.97 (-0.46, 2.41) | 1.16 (-0.41, 2.73) |
| 7-day mean PM_2.5_^d^ | 0.27 (-1.25, 1.79) | 0.22 (-1.43, 1.88) |
| 21-day mean PM_2.5_^e^ | 0.78 (-1.06, 2.63) | 0.52 (-1.65, 2.71) |
| **Blood Pressure** | | |
| ***Systolic blood pressure*** |  |  |
| Same-day PM_2.5_^b^ | -0.14 (-0.73, 0.45) | -0.10 (-0.75, 0.54) |
| 3-day mean PM_2.5_^c^ | -0.67 (-1.44, 0.10) | -0.95 (-1.86, -0.05) |
| 7-day mean PM_2.5_^d^ | -0.76 (-1.61, 0.09) | -1.11 (-2.12, -0.09) |
| 21-day mean PM_2.5_^e^ | -0.90 (-1.90, 0.10) | -1.70 (-2.98, -0.41) |
| ***Diastolic blood pressure*** |  |  |
| Same-day PM_2.5_^b^ | -0.07 (-0.56, 0.41) | -0.16 (-0.68, 0.36) |
| 3-day mean PM_2.5_^c^ | -0.23 (-0.87, 0.42) | -0.44 (-1.20, 0.30) |
| 7-day mean PM_2.5_^d^ | -0.07 (-0.80, 0.65) | -0.27 (-1.13, 0.59) |
| 21-day mean PM_2.5_^e^ | 0.05 (-0.80, 0.89) | -0.24 (-1.33, 0.84) |

N=344 measurements for retinal vessel diameter analyses, N=432 measurements for blood pressure analyses. 5 μg/m^3^ is the approximate interquartile range of PM_2.5_

^a^ Adjusted for 7-day mean temperature and humidity, body mass index-for-age z-score on the day of the retinal image, sex, age (years), maternal education (high school or less vs. community/technical college vs. university), and time of day of outcome assessment (≤11:00 AM vs. >11:00 AM).

^b^ PM_2.5_ on the day of the outcome assessment

^c^ Mean PM_2.5_ on the day of the outcome assessment and two preceding days

^d^ Mean PM_2.5_ on the day of the outcome assessment and 6 preceding days

^e^ Mean PM_2.5_ on the day of the outcome assessment and 20 preceding days

**Table S3:** Estimated change and 95% confidence interval in retinal blood vessel diameter (μm) and blood pressure (mm Hg) associated with 10 ppb increase in O_x_

|  | **Crude** | **Adjusted^a^** |
| --- | --- | --- |
| **Retinal Blood Vessel Diameter** | | |
| ***Central retinal arteriolar diameter*** | |  |
| Same-day O_x_^b^ | -1.10 (-2.48, 0.28) | -1.78 (-3.27, -0.28) |
| 3-day mean O_x_^c^ | -0.97 (-2.66, 0.72) | -1.99 (-3.92, -0.05) |
| 7-day mean O_x_^d^ | -1.59 (-3.41, 0.22) | -2.63 (-4.63, -0.63) |
| 21-day mean O_x_^e^ | -1.88 (-3.93, 0.17) | -2.56 (-4.71, -0.41) |
| ***Central retinal venular diameter*** | |  |
| Same-day O_x_^b^ | -0.44 (-2.05, 1.16) | -0.38 (-2.10, 1.34) |
| 3-day mean O_x_^c^ | -0.83 (-2.80, 1.13) | -0.83 (-3.04, 1.38) |
| 7-day mean O_x_^d^ | -0.47 (-2.60, 1.66) | -0.63 (-2.94, 1.67) |
| 21-day mean O_x_^e^ | -0.64 (-3.08, 1.79) | -0.74 (-3.21, 1.74) |
| **Blood Pressure** | | |
| ***Systolic blood pressure*** |  |  |
| Same-day O_x_^b^ | 0.74 (-0.28, 1.77) | 0.39 (-0.78, 1.58) |
| 3-day mean O_x_^c^ | 1.31 (0.10, 2.51) | 1.13 (-0.37, 2.64) |
| 7-day mean O_x_^d^ | 1.51 (0.24, 2.78) | 1.23 (-0.33, 2.79) |
| 21-day mean O_x_^e^ | 1.81 (0.39, 3.22) | 1.59 (-0.06, 3.25) |
| ***Diastolic blood pressure*** |  |  |
| Same-day O_x_^b^ | -0.26 (-1.10, 0.59) | -0.23 (-1.19, 0.72) |
| 3-day mean O_x_^c^ | -0.16 (-1.16, 0.85) | -0.06 (-1.28, 1.16) |
| 7-day mean O_x_^d^ | 0.06 (-1.03, 1.14) | 0.16 (-1.13, 1.45) |
| 21-day mean O_x_^e^ | 0.28 (-0.95, 1.52) | 0.45 (-0.95, 1.85) |

N=344 measurements for retinal vessel diameter analyses, N=432 measurements for blood pressure analyses. 10 ppb is the approximate interquartile range of O_x_

^a^ Adjusted for 7-day mean temperature and humidity, body mass index-for-age z-score on the day of the retinal image, sex, age (years), maternal education (high school or less vs. community/technical college vs. university), and time of day of outcome assessment (≤11:00 AM vs. >11:00 AM).

^b^ O_x_ on the day of the outcome assessment

^c^ Mean O_x_ on the day of the outcome assessment and two preceding days

^d^ Mean O_x_ on the day of the outcome assessment and 6 preceding days

^e^ Mean O_x_ on the day of the outcome assessment and 20 preceding days

**Table S4:** Estimated change and 95% confidence interval in retinal blood vessel diameter (μm) and blood pressure (mm Hg) associated with 5 μg/m^3^ increase in PM_2.5_, excluding days in which PM_2.5_ was imputed

|  | **N** | **Crude** | **Adjusted^a^** |
| --- | --- | --- | --- |
| **Retinal Blood Vessel Diameter** | | | |
| ***Central retinal arteriolar equivalent*** | | |  |
| Same-day PM_2.5_^b^ | 276 | -0.17 (-1.08, 0.74) | 0.03 (-0.93, 0.99) |
| 3-day mean PM_2.5_^c^ | 268 | 0.02 (-1.26, 1.30) | 0.45 (0.98, 1.89) |
| 7-day mean PM_2.5_^d^ | 254 | 0.46 (-0.94, 1.85) | 0.97 (-0.62, 2.56) |
| 21-day mean PM_2.5_^e^ | 231 | 0.71 (-0.86, 2.29) | 1.81 (-0.14, 3.76) |
| ***Central retinal venular equivalent*** | | |  |
| Same-day PM_2.5_^b^ | 276 | 0.31 (-0.72, 1.35) | 0.13 (-0.92, 1.19) |
| 3-day mean PM_2.5_^c^ | 268 | 0.82 (-0.73, 2.36) | 0.88 (-0.81, 2.57) |
| 7-day mean PM_2.5_^d^ | 254 | 0.82 (-1.01, 2.65) | 1.00 (-1.06, 3.05) |
| 21-day mean PM_2.5_^e^ | 231 | 0.64 (-1.43, 2.72) | 1.09 (-1.50, 3.67) |
| **Blood Pressure** | | | |
| ***Systolic blood pressure*** | |  |  |
| Same-day PM_2.5_^b^ | 357 | -0.17 (-0.79, 0.45) | -0.13 (-0.81, 0.54) |
| 3-day mean PM_2.5_^c^ | 349 | -0.55 (-1.37, 0.26) | -0.73 (-1.69, 0.24) |
| 7-day mean PM_2.5_^d^ | 334 | -0.71 (-1.63, 0.21) | -1.03 (-2.18, 0.11) |
| 21-day mean PM_2.5_^e^ | 313 | -0.82 (-1.90, 0.26) | -1.43 (-2.87, 0.00) |
| ***Diastolic blood pressure*** | |  |  |
| Same-day PM_2.5_^b^ | 357 | -0.11 (-0.60, 0.38) | -0.22 (-0.75, 0.30) |
| 3-day mean PM_2.5_^c^ | 349 | -0.21 (-0.86, 0.44) | -0.46 (-1.22, 0.31) |
| 7-day mean PM_2.5_^d^ | 334 | -0.12 (-0.86, 0.61) | -0.45 (-1.36, 0.46) |
| 21-day mean PM_2.5_^e^ | 313 | 0.06 (-0.80, 0.92) | -0.23 (-1.39, 0.92) |

5 μg/m^3^ is the approximate interquartile range of PM_2.5_

^a^ Adjusted for 7-day mean temperature and humidity, body mass index-for-age z-score on the day of the retinal image, sex, age (years), maternal education (high school or less vs. community/technical college vs. university), and time of day of outcome assessment (≤11:00 AM vs. >11:00 AM).

^b^ PM_2.5_ on the day of the outcome assessment

^c^ Mean PM_2.5_ on the day of the outcome assessment and two preceding days

^d^ Mean PM_2.5_ on the day of the outcome assessment and 6 preceding days

^e^ Mean PM_2.5_ on the day of the outcome assessment and 20 preceding days

**Table S5:** Estimated change and 95% confidence interval in retinal blood vessel diameter (μm) and blood pressure (mm Hg) associated with 15 ppb increase in O_3_

|  | **Crude** | **Adjusted^a^** |
| --- | --- | --- |
| **Retinal Blood Vessel Diameter** | | |
| ***Central retinal arteriolar diameter*** | |  |
| Same-day O_3_^b^ | -1.02 (-2.31, 0.27) | -1.61 (-3.01, -0.21) |
| 3-day mean O_3_^c^ | -0.94 (-2.54, 0.66) | -1.89 (-3.74, -0.05) |
| 7-day mean O_3_^d^ | -1.61 (-3.33, 0.12) | -2.57 (-4.48, -0.66) |
| 21-day mean O_3_^e^ | -1.83 (-3.80, 0.14) | -2.45 (-4.53, -0.38) |
| ***Central retinal venular diameter*** | |  |
| Same-day O_3_^b^ | -0.54 (-2.04, 0.96) | -0.45 (-2.05, 1.16) |
| 3-day mean O_3_^c^ | -0.92 (-2.78, 0.94) | -0.87 (-2.98, 1.24) |
| 7-day mean O_3_^d^ | -0.60 (-2.63, 1.43) | -0.65 (-2.85, 1.55) |
| 21-day mean O_3_^e^ | -0.76 (-3.10, 1.57) | -0.74 (-3.12, 1.64) |
| **Blood Pressure** | | |
| ***Systolic blood pressure*** |  |  |
| Same-day O_3_^b^ | 0.65 (-0.32, 1.61) | 0.35 (-0.76, 1.46) |
| 3-day mean O_3_^c^ | 1.22 (0.07, 2.36) | 1.13 (-0.31, 2.58) |
| 7-day mean O_3_^d^ | 1.40 (0.18, 2.61) | 1.19 (-0.32, 2.70) |
| 21-day mean O_3_^e^ | 1.71 (0.34, 3.07) | 1.59 (-0.02, 3.19) |
| ***Diastolic blood pressure*** |  |  |
| Same-day O_3_^b^ | -0.25 (-1.05, 0.54) | -0.24 (-1.13, 0.66) |
| 3-day mean O_3_^c^ | -0.16 (-1.11, 0.79) | -0.06 (-1.23, 1.11) |
| 7-day mean O_3_^d^ | 0.03 (-1.00, 1.07) | 0.15 (-1.10, 1.39) |
| 21-day mean O_3_^e^ | 0.24 (-0.94, 1.43) | 0.42 (-0.94, 1.79) |

N=344 measurements for retinal vessel diameter analyses, N=432 measurements for blood pressure analyses. 15 ppb is the approximate interquartile range of O_3_

^a^ Adjusted for 7-day mean temperature and humidity, body mass index-for-age z-score on the day of the retinal image, sex, age (years), maternal education (high school or less vs. community/technical college vs. university), and time of day of outcome assessment (≤11:00 AM vs. >11:00 AM).

^b^ O_3_ on the day of the outcome assessment

^c^ Mean O_3_ on the day of the outcome assessment and two preceding days

^d^ Mean O_3_ on the day of the outcome assessment and 6 preceding days

^e^ Mean O_3_ on the day of the outcome assessment and 20 preceding days

**Table S6:** Estimated change and 95% confidence interval in retinal blood vessel diameter (μm) and blood pressure (mm Hg) associated with 2 ppb increase in NO_2_

|  | **Crude** | **Adjusted^a^** |
| --- | --- | --- |
| **Retinal Blood Vessel Diameter** | | |
| ***Central retinal arteriolar diameter*** | |  |
| Same-day NO_2_^b^ | 0.34 (-0.56, 1.25) | 0.37 (-0.71, 1.44) |
| 3-day mean NO_2_^c^ | 0.53 (-0.63, 1.70) | 0.94 (-0.88, 2.77) |
| 7-day mean NO_2_^d^ | 1.17 (-0.11, 2.45) | 2.80 (0.58, 5.02) |
| 21-day mean NO_2_^e^ | 0.77 (-0.81, 2.34) | 1.40 (-1.31, 4.11) |
| ***Central retinal venular diameter*** | |  |
| Same-day NO_2_^b^ | 1.08 (0.04, 2.13) | 0.99 (-0.22, 2.21) |
| 3-day mean NO_2_^c^ | 1.29 (-0.06, 2.64) | 1.48 (-0.60, 3.56) |
| 7-day mean NO_2_^d^ | 1.33 (-0.16, 2.83) | 1.37 (-1.19, 3.93) |
| 21-day mean NO_2_^e^ | 1.47 (-0.36, 3.30) | 1.23 (-1.87, 4.33) |
| **Blood Pressure** | | |
| ***Systolic blood pressure*** |  |  |
| Same-day NO_2_^b^ | 0.08 (-0.56, 0.73) | 0.04 (-0.76, 0.84) |
| 3-day mean NO_2_^c^ | -0.21 (-1.03, 0.61) | -0.97 (-2.29, 0.34) |
| 7-day mean NO_2_^d^ | 0.06 (-0.89, 1.02) | -0.46 (-2.15, 1.22) |
| 21-day mean NO_2_^e^ | -0.12 (-1.26, 1.02) | -1.36 (-3.33, 0.60) |
| ***Diastolic blood pressure*** |  |  |
| Same-day NO_2_^b^ | 0.18 (-0.35, 0.70) | 0.20 (-0.44, 0.85) |
| 3-day mean NO_2_^c^ | 0.14 (-0.54, 0.83) | 0.07 (-1.00, 1.13) |
| 7-day mean NO_2_^d^ | 0.15 (-0.65, 0.94) | 0.05 (-1.33, 1.43) |
| 21-day mean NO_2_^e^ | 0.20 (-0.75, 1.15) | 0.07 (-1.52, 1.66) |

N=344 measurements for retinal vessel diameter analyses, N=432 measurements for blood pressure analyses. 2 ppb is the approximate interquartile range of NO_2_

^a^ Adjusted for 7-day mean temperature and humidity, body mass index-for-age z-score on the day of the retinal image, sex, age (years), maternal education (high school or less vs. community/technical college vs. university), and time of day of outcome assessment (≤11:00 AM vs. >11:00 AM).

^b^ NO_2_ on the day of the outcome assessment

^c^ Mean NO_2_ on the day of the outcome assessment and two preceding days

^d^ Mean NO_2_ on the day of the outcome assessment and 6 preceding days

^e^ Mean NO_2_ on the day of the outcome assessment and 20 preceding days

**Table S7:** Estimated change and 95% confidence interval in retinal blood vessel diameter (μm) and blood pressure (mm Hg) associated with 5 μg/m^3^ increase in PM_2.5_, in models additionally adjusted for season

|  | **Adjusted^a^** |
| --- | --- |
| **Retinal Blood Vessel Diameter** | |
| ***Central retinal arteriolar diameter*** | |
| Same-day PM_2.5_^b^ | 0.05 (-0.92, 1.01) |
| 3-day mean PM_2.5_^c^ | 0.56 (-1.13, 2.26) |
| 7-day mean PM_2.5_^d^ | 1.33 (-0.40, 3.06) |
| 21-day mean PM_2.5_^e^ | 1.95 (-0.26, 4.15) |
| ***Central retinal venular diameter*** | |
| Same-day PM_2.5_^b^ | 0.35 (-0.74, 1.44) |
| 3-day mean PM_2.5_^c^ | 1.53 (-0.40, 3.46) |
| 7-day mean PM_2.5_^d^ | 0.12 (-1.87, 2.11) |
| 21-day mean PM_2.5_^e^ | 0.41 (-2.13, 2.94) |
| **Blood Pressure** | |
| ***Systolic blood pressure*** |  |
| Same-day PM_2.5_^b^ | 0.04 (-0.64, 0.72) |
| 3-day mean PM_2.5_^c^ | -0.84 (-1.86, 0.19) |
| 7-day mean PM_2.5_^d^ | -0.95 (-2.11, 0.16) |
| 21-day mean PM_2.5_^e^ | -1.62 (-3.03, -0.20) |
| ***Diastolic blood pressure*** |  |
| Same-day PM_2.5_^b^ | -0.14 (-0.69, 0.41) |
| 3-day mean PM_2.5_^c^ | -0.45 (-1.30, 0.39) |
| 7-day mean PM_2.5_^d^ | -0.22 (-1.17, 0.72) |
| 21-day mean PM_2.5_^e^ | -0.23 (-1.40, 0.94) |

N=344 measurements for retinal vessel diameter analyses, N=432 measurements for blood pressure analyses. 5 μg/m^3^ is the approximate interquartile range of PM_2.5_

^a^ Adjusted for 7-day mean temperature and humidity, body mass index-for-age z-score on the day of the retinal image, sex, age (years), maternal education (high school or less vs. community/technical college vs. university), and time of day of outcome assessment (≤11:00 AM vs. >11:00 AM), and season (September-November/December-February/March-May/June).

^b^ PM_2.5_ on the day of the outcome assessment

^c^ Mean PM_2.5_ on the day of the outcome assessment and two preceding days

^d^ Mean PM_2.5_ on the day of the outcome assessment and 6 preceding days

^e^ Mean PM_2.5_ on the day of the outcome assessment and 20 preceding days

**Table S8:** Estimated change and 95% confidence interval in retinal blood vessel diameter (μm) and blood pressure (mm Hg) associated with 10 ppb increase in O_x_, in models additionally adjusted for season

|  | **Adjusted^a^** |
| --- | --- |
| **Retinal Blood Vessel Diameter** | |
| ***Central retinal arteriolar diameter*** | |
| Same-day O_x_^b^ | -2.05 (-3.71, -0.38) |
| 3-day mean O_x_^c^ | -2.38 (-4.56, -0.20) |
| 7-day mean O_x_^d^ | -3.58 (-5.94, -1.22) |
| 21-day mean O_x_^e^ | -4.87 (-7.78, -1.96) |
| ***Central retinal venular diameter*** | |
| Same-day O_x_^b^ | -0.15 (-2.06, 1.76) |
| 3-day mean O_x_^c^ | -0.53 (-3.02, 1.96) |
| 7-day mean O_x_^d^ | -0.33 (-3.04, 2.39) |
| 21-day mean O_x_^e^ | -0.83 (-4.19, 2.53) |
| **Blood Pressure** | |
| ***Systolic blood pressure*** |  |
| Same-day O_x_^b^ | 0.12 (-1.20, 1.44) |
| 3-day mean O_x_^c^ | 0.90 (-0.80, 2.60) |
| 7-day mean O_x_^d^ | 0.95 (-0.92, 2.81) |
| 21-day mean O_x_^e^ | 1.59 (-0.73, 3.91) |
| ***Diastolic blood pressure*** |  |
| Same-day O_x_^b^ | -0.41 (-1.47, 0.65) |
| 3-day mean O_x_^c^ | -0.22 (-1.58, 1.14) |
| 7-day mean O_x_^d^ | 0.03 (-1.48, 1.53) |
| 21-day mean O_x_^e^ | 0.49 (-1.40, 2.37) |

N=344 measurements for retinal vessel diameter analyses, N=432 measurements for blood pressure analyses. 10 ppb is the approximate interquartile range of O_x_

^a^ Adjusted for 7-day mean temperature and humidity, body mass index-for-age z-score on the day of the retinal image, sex, age (years), maternal education (high school or less vs. community/technical college vs. university), and time of day of outcome assessment (≤11:00 AM vs. >11:00 AM), and season (September-November/December-February/March-May/June).

^b^ O_x_ on the day of the outcome assessment

^c^ Mean O_x_ on the day of the outcome assessment and two preceding days

^d^ Mean O_x_ on the day of the outcome assessment and 6 preceding days

^e^ Mean O_x_ on the day of the outcome assessment and 20 preceding days


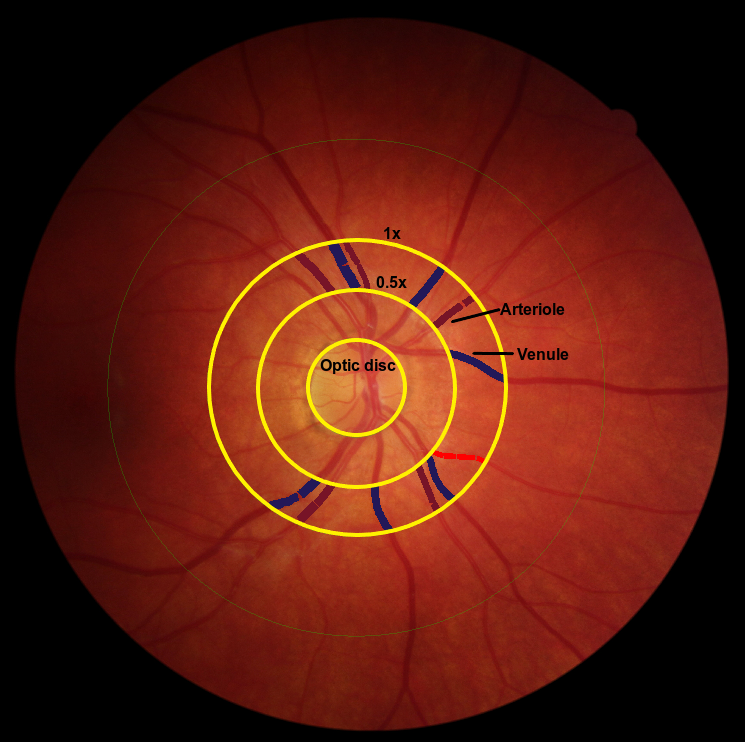


**Figure S1:** Central retinal arteriolar equivalent (CRAE) and central retinal venular equivalent (CRVE) calculated within an area equal to 0.5-1 times the disc diameter from the optic disc margin. Arterioles are identified in red and venules in blue.


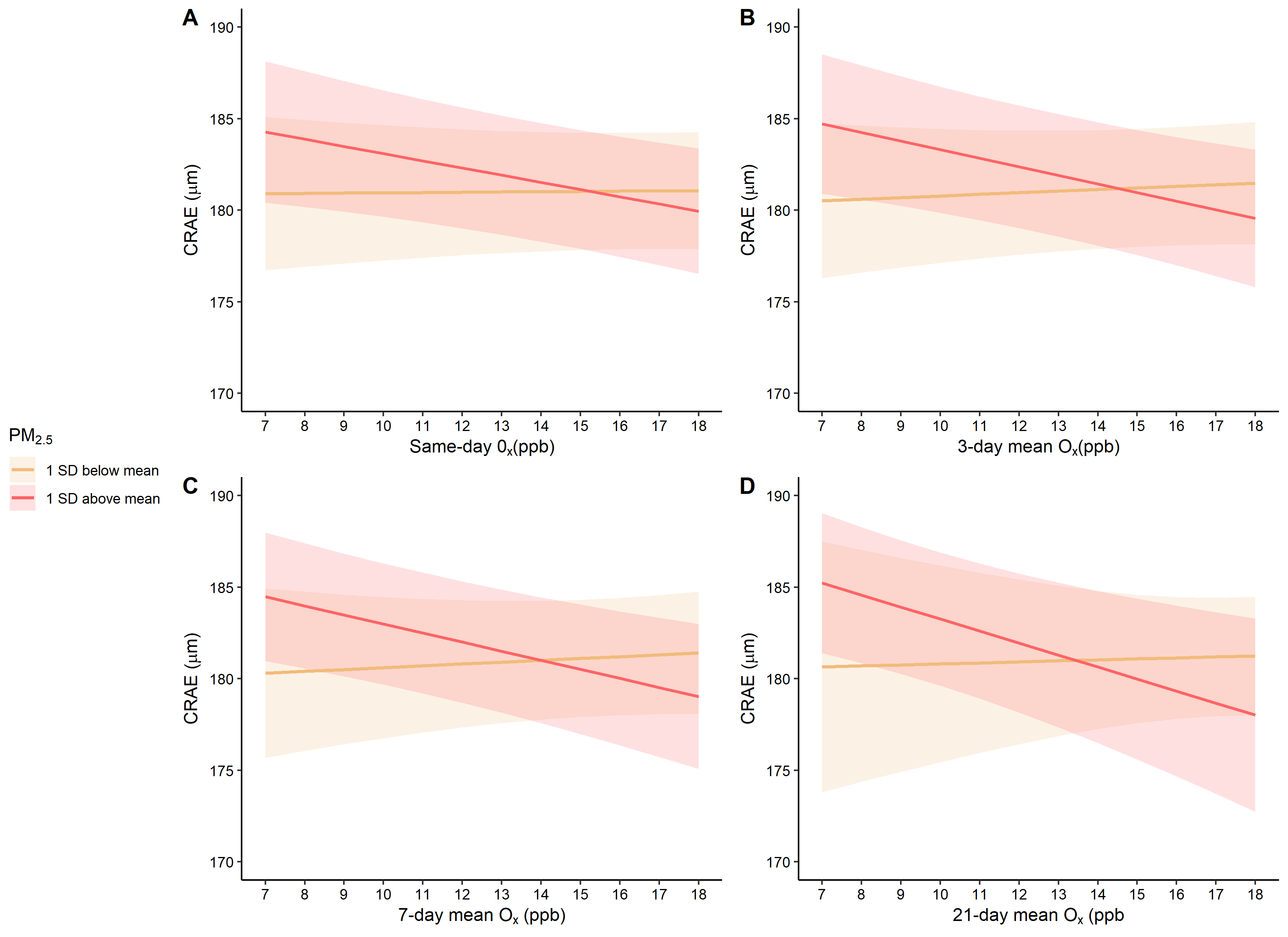


**Figure S2:** Predicted values and 95% CIs for central retinal arteriolar equivalent (CRAE) at different concentrations of O_x_ (7-18 ppb), stratified by PM_2.5_ (1 standard deviation below and above mean PM_2.5_ concentrations). Plots correspond to (A): Same-day exposure lag; (B): 3-day mean exposure lag; (C): 7-day mean exposure lag; (D): 21-day mean exposure lag.


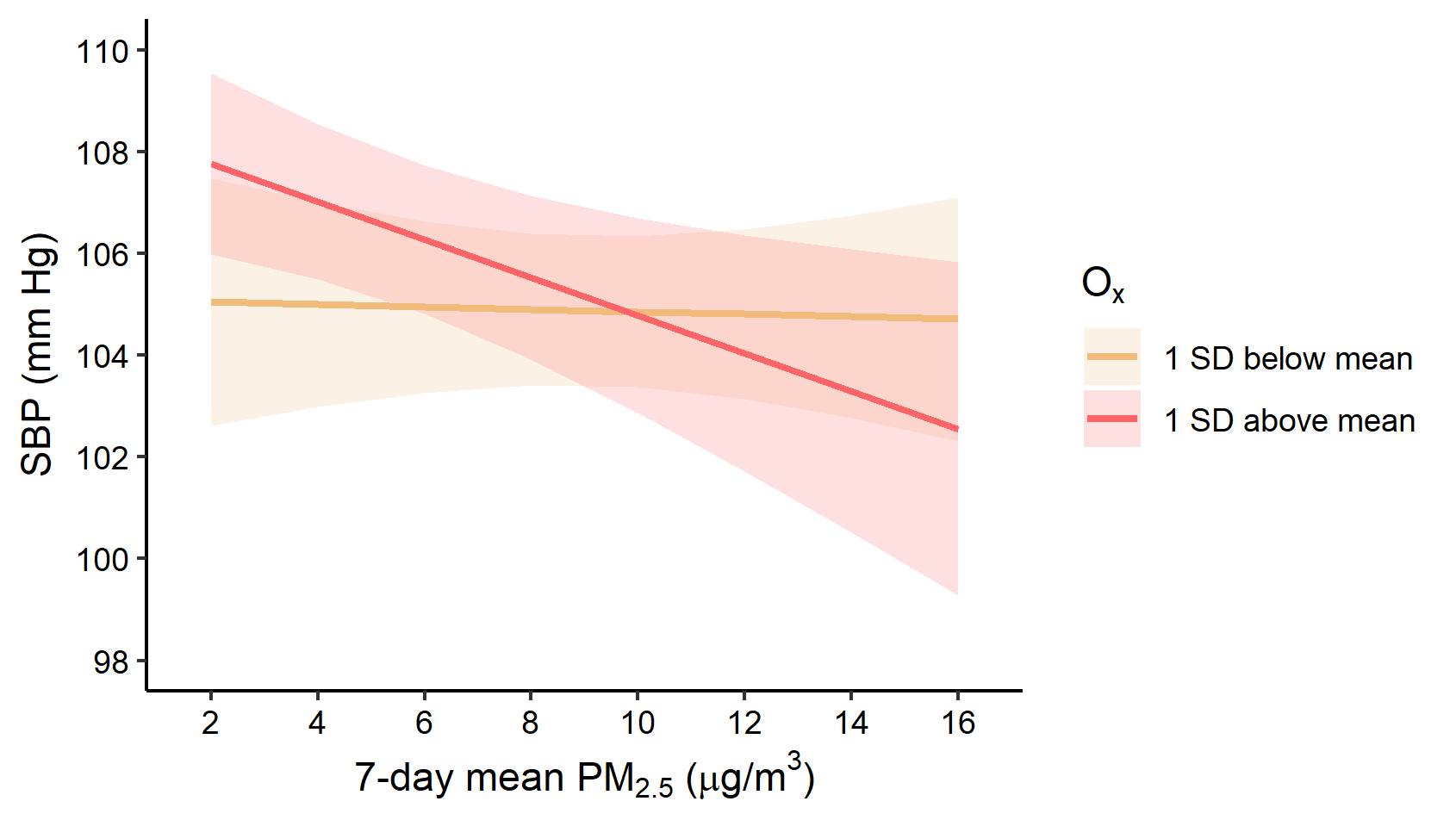


**Figure S3**: Predicted values and 95% CIs for systolic blood pressure at different concentrations of 7-day mean PM_2.5_, stratified by 7-day mean O_x_ (1 standard deviation below and above 7-day mean O_x_ concentrations).
